# Exploring longitudinal shifts in international nurse migration to the United States between 2003 and 2013 through a random effects panel data analysis

**DOI:** 10.1186/s12960-016-0118-7

**Published:** 2016-06-30

**Authors:** Allison Squires, Melissa T. Ojemeni, Simon Jones

**Affiliations:** New York University College of Nursing, 433 First Avenue, New York, NY 10010 USA; New York University-School of Medicine-Population Health, 227 East 30th Street, New York, 10016 New York United States

**Keywords:** Internationally-educated nurses, Nursing credentialing and licensure examination for registered nurses, Nurse migration, United States, WHO code

## Abstract

**Background:**

No study has examined the longitudinal trends in National Council Licensure Exam for Registered Nurse (NCLEX-RN) applicants and pass rates among internationally-educated nurses (IENs) seeking to work in the United States, nor has any analysis explored the impact of specific events on these trends, including changes to the NCLEX-RN exam, the role of the economic crisis, or the passing of the WHO Code on the International Recruitment of Health Personnel. This study seeks to understand the impact of the three aforementioned factors that may be influencing current and future IEN recruitment patterns in the United States.

**Methods:**

In this random effects panel data analysis, we analyzed 11 years (2003–2013) of annual IEN applicant numbers and pass rates for registered nurse credentialing. Data were obtained from publicly available reports on exam pass rates. With the global economic crisis and NCLEX-RN changes in 2008 coupled with the WHO Code passage in 2010, we sought to compare if (1) the number of applicants changed significantly after those 2 years and (2) if pass rates changed following exam modifications implemented in 2008 and 2011.

**Results:**

A total of 177 countries were eligible for inclusion in this analysis, representing findings from 200,453 IEN applicants to the United States between 2003 and 2013. The majority of applicants were from the Philippines (58 %) and India (11 %), with these two countries combined representing 69 % of the total. Candidates from Sub-Saharan African countries totalled 7133 (3 % of all applications) over the study period, with half of these coming from Nigeria alone. No significant changes were found in the number of candidates following the 2008 economic crisis or the 2010 WHO Code, although pass rates decreased significantly following the 2008 exam modifications and the WHO Code implementation.

**Conclusion:**

This study suggests that, while the WHO Code has had an influence on overall IEN migration dynamics to the United States by decreasing candidate numbers, in most cases, the WHO Code was not the single cause of these fluctuations. Indeed, the impact of the NCLEX-RN exam changes appears to exert a larger influence.

## Background

Twenty first century international-educated nurse (IEN) migration has exploded in many ways due to the profession’s ability to provide a tangible and transferable skill set in almost any country. According to the OECD and the United Nations, the number of persons migrating has increased from an estimated 150 million in 2000 to 231.5 million in 2013, with a recent increase in the migration of skilled workers such as nurses [1].

Healthcare is labour intensive and the availability of sufficient, well trained and motivated staff is key to the effectiveness of healthcare services delivery [2]. Fuelled primarily by aging populations, an increase in co-morbid conditions and the use of technology, the need for healthcare services and nurses will continue to grow as projections point to reductions in the supply of nurses in some countries while others will continue to suffer under current shortages [3]. Eighty three countries – 81 of which are low- and middle-income countries (LMICs), fall below the recommended 22.8 skilled health workers (nurses, midwives and physicians) per 100,000 population [4]. If this trend continues, there will be a global shortage of 12.9 million nurses, midwives, and physicians by 2035 [4].^1^ International recruitment of nurses, therefore, will remain an option to help address shortages.

In response to the ongoing migration trends and subsequent health systems weakening due to staff shortages, the World Health Assembly, the legislative body of WHO, set out in 2004 to create a code of practice on ethical recruitment. The WHO Global Code of Practice on the International Recruitment of Health Personnel, hereafter WHO Code, became the first globally applicable and universally accepted regulatory framework for national and subnational actors involved in international health workforce recruitment [5, 6]. In essence, the WHO Code has 10 articles and encourages member states to create a sustainable health workforce through planning, educating, training and retaining, such that their need to recruit migrant workers is reduced, particularly from ‘do not recruit’ countries who suffer from critical provider shortages [6, 7].

One regulatory strategy to manage migration flows is a professional licensure exam. These examinations help determine whether a recent graduate from a professional training school has the requisite knowledge and critical thinking abilities to safely practice. For IENs, these examinations help determine whether the international candidate meets the same requirements as local candidates. In the United States, IENs are required to pass the Nursing Credentialing and Licensure Examination for Registered Nurses (NCLEX-RN). Every 3 years, the exam changes as practice and psychometric analyses further refine what represents a demonstration by the test taker of their baseline clinical knowledge, its application to safe clinical practice, critical thinking skills, and health system knowledge [7]. Major content changes can precipitate decreases in pass rates for domestic and international candidates. Changes to the test were implemented in 2005, 2008, 2011, and 2014, and recent changes have included an increased number of questions on pathophysiology, laboratory test interpretation, and delegation of patient care tasks. The changes made in 2008 and 2011 are both significant for the present study due to the economic crisis of 2008 and the passing of the WHO Code in late 2010.

To date, no study has examined the longitudinal trends in the NCLEX-RN among international applicants and their pass rates nor has any analysis explored the impact of specific events on these trends, including changes to the NCLEX-RN exam, the role of economic crises, or the passing of the WHO Code. With human resources for health data quality improving globally, these types of analyses are now possible. Therefore, we sought to understand the influences of the three aforementioned factors and their potential implications on current and future registered nurse recruitment patterns in the United States, as well as to explore the methodological issues that may arise from analysing longitudinal international nurse migration data before and after the implementation of the WHO Code. Since the United States has historically been a top receiving country of IENs [8] and given the good quality data available regarding IENs, it provides a good test case for this type of analysis. The quality of the data also allows examination of the intersections of global economics, the influence of the WHO Code, and the role of the NCLEX-RN exam on IEN migration patterns.

The present study is timely given the shift in United States nursing labour market dynamics since 2008 due to a variety of factors. The global economic crash in the autumn of 2008 meant that, by 2009, hiring patterns among healthcare facilities had drastically changed. New graduate nurses, previously graduating with a job or at least several offers, often had to wait 6 months or more to obtain their first clinical position [9–11]. Layoffs in non-health sectors also meant that several people sought career changes, with nursing being a popular career option. Nursing schools, already at peak enrolments, saw applications increase significantly between 2008 and 2013 [12, 13]. By 2011, nursing labour market production had reached a production equilibrium, with enough nurses under 30 graduating from nursing schools to replace those who would retire [14, 15]. Nonetheless, recent projections from the United States Department of Health and Human Services suggest that shortages will persist, but only in selected states concentrated in the middle of the country [15].

International recruitment of nurses to the United States also began to decrease after the economic crisis as the United States IEN recruitment industry was decimated [16]. The passage of the WHO Code, in late 2010, further punctuated changes in international recruitment patterns. Work visas for IENs also decreased and new English language competency testing requirements made attaining a visa for migrant nurses even more difficult [17].

## Methods

For the purposes of this paper, we posit that the number of applicants for the NCLEX-RN exam is an indicator of the level of interest in IENs seeking work in the United States and may also be a proxy indicator of the nurses’ home country domestic labour market conditions, which are sensitive to a country’s economic situation [16]. An individual candidate’s likelihood of passing the exam can reflect multiple explanations, including quality of the individual candidate, quality of nursing education in their home country, and changes to the exam made by the National Council of State Boards of Nursing (NCSBN) in the United States. The latter is important because it also affects domestic pass rates on the exam, which often decrease following the implementation of changes. Finally, both the 2008 economic crisis and late 2010 passage of the WHO Code are key events that may have affected IEN migration to the United States.

For this random effects panel data analysis, we analyzed 11 years (2003–2013) of annual IEN applicant numbers and pass rates for registered nurse credentialing. As part of an ongoing project and at the time of submission, 11 years was as far as the statistical analysis was completed. It is our hope to be able to provide a longer period of analysis in the future. In addition, it is important to note that the number of successful testing candidates will be higher than the number of IENs actually working due to visa processing issues that delay entry into the labour market. We obtained data from publicly available reports on exam pass rates available from the website of the NCSBN. To be included in the study, countries had to have at least one applicant between 2003 and 2013.

An initial dataset was created in a standard spreadsheet program. Variables included the country of origin of the testing candidate, English as an official language of a country, gross national income based on purchasing power parity (GNI-PPP), national health expenditure as a percentage of GDP, and the passage of the WHO Code were included in the model.

Since the NCLEX-RN is offered in English and language skills are known to affect pass rates on the exam when English is not the test taker’s first language [18–20], countries were categorized as either English as the official language, English as one of several official languages, and English not as an official language. Classification of English language status of a country was derived from the United States CIA.gov website of country profiles. Including this variable would help determine the role of language skills in driving IEN migration or as a potential confounder, among other factors.

Health expenditure as a percentage of GDP and GNI-PPP were both included to account for domestic health labour market and economic conditions. Health expenditure as a percentage of GDP provides a proxy for labour expenditures, of which nurses will represent the largest expense in most countries. The GNI-PPP was used for an overall individual economic indicator as a proxy for economic stability and the ability of a nurse to obtain the financial resources necessary to migrate to the United States for work.

A further variable that would affect the analysis was the fact that, if no candidates from a given country applied in a specific year, the pass rate would also be zero since statistical software may read ‘0’ as missing data. Multiple ‘0’ results would also have the potential to skew the results. Finally, analyses had to be adjusted for the number of candidates from the Philippines to the United States.

We then conducted two sets of analyses. In the first set, we examined descriptive trends in the data over the 11-year period and were able to complete this by country and region. We then used the years 2008 and 2010 as key ‘event’ years in the analysis. In 2008, there was global economic crisis in the latter part of the year and the NCLEX-RN exam made a major change in format and content. In late 2010, the WHO Code was passed and therefore potential changes in applicant profiles in the succeeding years would be anticipated. Thus, we sought to compare if (1) the number of applicants and (2) pass rates changed significantly after 2008 and 2011. We conducted the second set of analyses through a random effects panel data approach, which was fitted to predict the pass rate and number of candidates based on the aforementioned variables using the PLM package that is part of R statistical software [19]. The method described by Croissant and Millo [21] was applied to the fitting approach.

## Results

A total of 177 countries were eligible for inclusion in this analysis, representing data from 200,453 IEN applicants to the United States between 2003 and 2013. Of those applicants, 58 % were from the Philippines and 11 % from India. Combined, these two countries represented 69 % of the total IEN applicants to the United States. Sub-Saharan African candidates totalled 7133 (3 % of all candidates) over the study period, with half of those coming from Nigeria alone. Among the other countries, 46 WHO ‘do not recruit’ countries were on the list.

The peak year for IEN applications to the United States was 2007, reflecting a significant increase from previous years, where growth in IEN applications had been more gradual. Since 2008, however, IEN applicant rates and the pass rates on the exam have steadily declined.

**Fig. 1:**
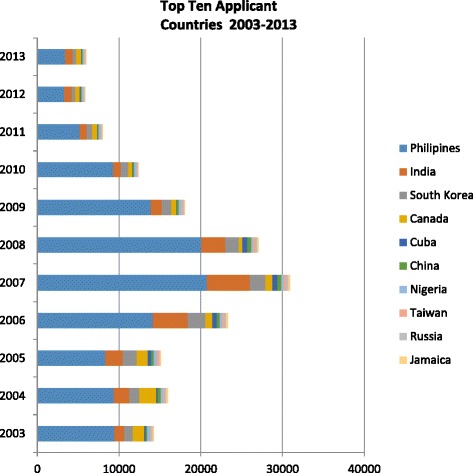
Top Ten Applicant Countries 2003-2013

**Fig. 2:**
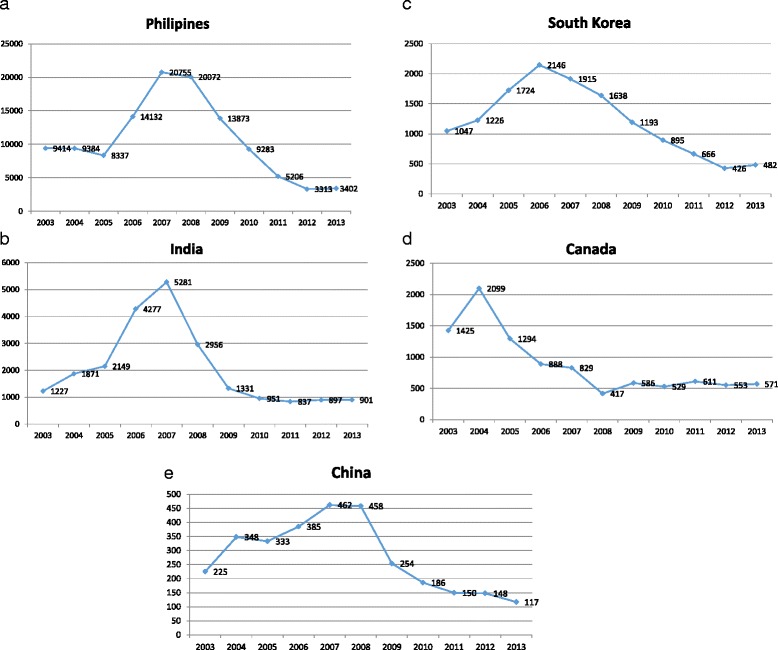
Top 5 Countries and Number of Candidates Taking the NCLEX-RN from 2003-2013

**Fig. 3:**
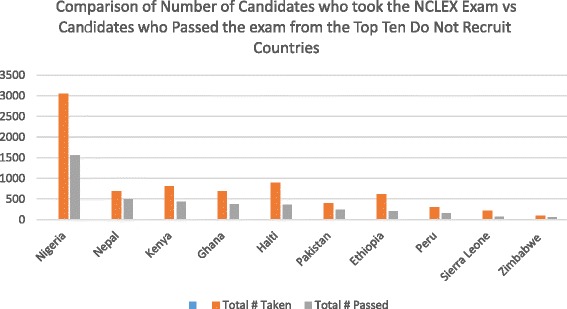
Comparison of Number of Candidates Who Took the NCLEX-RN Exam vs Candidates Who Passed the Exam from the Tope Ten Do Not Recruit Countries

Figure 1 illustrates the trends among the top 10 applicant countries to the United States from 2003 to 2013. Fig. 2a to e shows the number of applicants taking the NCLEX-RN exam from each of the top five countries over an 11-year period. While applicant trends continued to decrease over the 2 years following the passing of the WHO Code, data for 2013 suggests a recurring increase. Notably, however, applicant trends from most of the 46 ‘do not recruit’ countries continue to decline, even from high volume ‘sending countries’ like Nigeria. Fig. 3 provides a comparative assessment from the top 10 ‘do not recruit’ countries.

**Fig. 4:**
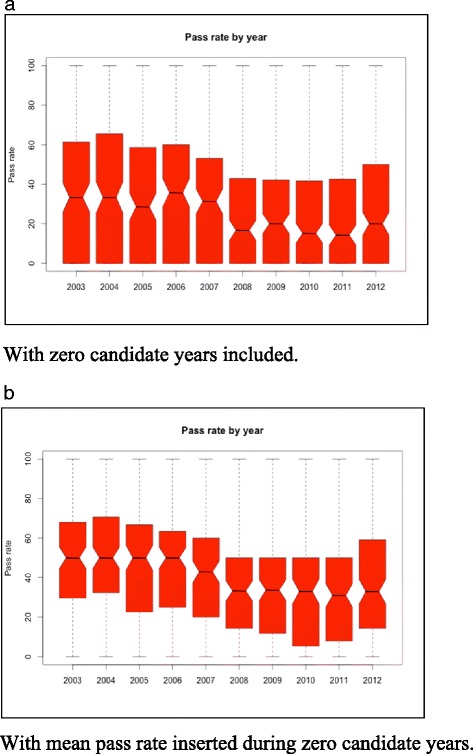
**a** Pass Rate by Year Wtih Zero Candidate Years Included. **b** Pass Rate by Year with Mean Pass Rate Inserted During Zero Candidate Year

Tables 1, 2 and 3 highlight the results from the random effects model. The variables had no effect on the overall number of candidates applying to take the exam even though the descriptive analyses show a downward trend. Exam pass rates, however, showed the opposite results; while historically averaging 50 %, these have averaged 28 % since 2008. Unsurprisingly, English language ability has had an effect on the ability to pass the NCLEX-RN exam (Table 2). Among the countries included in the study, 25 had English as the official language, 42 had English as one of several official languages, and 110 do did not have English as an official language. Candidates from countries where English was not an official language or one of several official languages had significantly lower exam pass rates, with differences from “English Only” countries at *P* = 0.001 and *P* = 0.002, respectively, and pass rates decreasing by 13–12 %. As mentioned above, the pass rate variable (Fig. 4a) was affected when there were no candidates from a given country in a given year. To check that this did not distort the result, we repeated the analysis using the mean pass rate for that country as (Fig. 4b) a sensible estimate for a missing value. This did not significantly change the results (Table 3).

**Table 1:** Random effects panel data analysis of variables on number of candidates

Effect	Estimate	LCI	UCI	SE	*t* value	*P* value
(Intercept: English only)	−735.366	−2230.798	760.066	762.990	−0.964	0.335
English not official	34.561	−396.728	465.850	220.050	0.157	0.875
English Multiple	−13.981	−514.891	486.930	255.571	−0.055	0.956
Healthcare expenditure	−21.625	−50.938	7.688	14.956	−1.446	0.148
GNI-PPP	39.559	−20.850	99.968	30.821	1.284	0.200
WHO Code	−12.950	−110.205	84.305	49.621	−0.261	0.794

GNI-PPP, Gross national income based on purchasing power parity; Lower Confidence Interval; Upper Confidence Interval; Standard Error

**Table 2:** Random effects panel data analysis on pass rates

Effect	Estimate	LCI	UCI	SE	*t* value	*P* value
(Intercept: English only)	−78.073	−106.48483	−49.660	14.496	−5.386	0.000
English not official	−12.965	−20.30369	−5.627	3.744	−3.463	0.001
English multiple	−13.454	−21.949441	−4.958	4.335	−3.104	0.002
Healthcare expenditure	0.901	0.03177809	1.770	0.443	2.032	0.042
GNI-PPP	4.658	3.52342418	5.792	0.579	8.048	0.000
WHO Code	−13.156	−17.598998	−8.712	2.267	−5.803	0.000

GNI-PPP, Gross national income based on purchasing power parity; Lower Confidence Interval; Upper Confidence Interval; Standard Error

**Table 3:** Random effects panel data analysis on pass rates with passing mean inserted in zero candidate years

Effect	Estimate	LCI	UCI	SE	*t* value	*P* value
(Intercept: English only)	−23.284	−56.264	9.696	16.827	−1.384	0.167
English not official	−7.090	−15.244	1.065	4.161	−1.704	0.089
English multiple	−12.913	−22.430	−3.396	4.856	−2.659	0.008
Healthcare expenditure	0.583	−0.430	1.596	0.517	1.128	0.259
GNI-PPP	2.700	1.391	4.009	0.668	4.044	0.000
WHO Code	−14.476	– 20.175	−8.778	2.907	−4.979	0.000

*GNI-PPP* Gross national income based on purchasing power parity; Lower Confidence Interval; Upper Confidence Interval; Standard Error

Overall, IENs had lower average pass rates on the NCLEX-RN exam compared to United States-educated nurses. In comparison to the IENs, first time, United States-educated nurse pass rates have not decreased significantly pre- and post-exam year changes. United States-educated nurses had a pass rate of 85.5 % and 87.3 % in 2004 and 2005, respectively, compared to 58.2 % and 58.1 % for IENs in the same years [22, 23]. Further, United States-educated nurses had a pass rate of 85.5 % and 86.7 % in 2007 and 2008, respectively, compared to 52 % and 45.3 % for IENs [24, 25]. Finally, United States-educated test takers had a pass rate of 87.4 % and 87.9 % in 2010 and 2011, respectively, compared to 38.6 % and 33.9 % for IENs [26, 27].

A country’s health expenditure was also observed to influence pass rates. With every 1 % increase in overall health expenditure, pass rates increased by 0.9 % (0.03 % to 1.7 %, *P* = 0.042). This was the only result in the model, however, that became non-significant once the variable of zero candidate’s in a year was corrected and, therefore, merits further exploration.

GNI-PPP’s effects were also observed to be significant for pass rates in both models, suggesting that a one unit increase in GNI-PPP will result in a 4.7 % (3.5–5.8 %, *P* < 0.0001) improvement in pass rates. This is a logical finding since nurses with more economic resources at their disposal would be able to obtain the necessary educational support to help them pass the exam.

Finally, while the 2008 economic crisis definitely impacted applicant rates, it appears that the 2008 change in the NCLEX-RN exam content may have had a greater impact. Following major content and format changes to the exam in 2008, pass rates by IENs dropped significantly. The trend continued and an additional drop was observed in 2011, although not as significant. Most notably, even the Philippines pass rate has dropped to 28 % as of 2014. Reference: https://www.ncsbn.org/15_2014_NCLEXExamStats_vol64.pdf .

## Discussion

The results from the present study suggest that the WHO Code has influenced recruitment patterns to the United States, especially influencing recruitment patterns from some ‘do not recruit’ countries. Overall, however, the event that appears to have had the most effect on IEN migration to the United States is the changes made to the NCLEX-RN exam implemented in 2008. In addition to an increased number of questions involving pathophysiology, laboratory test interpretation, and delegation of work in the United States context of care, the test item format also changed, with questions requiring multiple answers, ‘visual’ answers, and prioritization of responses. Questions shifted from the simpler, single answer multiple choice format to a more complex assessment of the test taker’s knowledge and critical thinking abilities. Thus, even candidates from strong ‘testing cultures’ and historically high pass rates on the exam experienced drops in pass rates. While specific country data is not yet available, overall, this trend has continued into 2014, with IEN pass rates now below 30 % for the first time and the overall number of candidates a mere 7790 [26].

So why would pathophysiology, lab test, and delegation content result in increased failure rates on the exam among IENs? The answer is rooted in problems with basic pathophysiology education and the significant variability of core science course education across nursing programs. Previous studies suggest that there can be multiple weaknesses in nursing education of IENs because of the variability between countries, with much of the difference being focused on pathophysiology [28–37]. Pathophysiology courses are often taught by non-nurses or physicians who may have their own ideas about what nurses need to know resulting, in the absence of teaching or educational standards, in poor basic pathophysiology knowledge and, likely, poor application in practice. Thus, IENs would need to have received good quality education in pathophysiology in order to pass the exam. While an individual might be able to overcome this knowledge deficit, this is unlikely without additional coursework.

Laboratory test interpretation is an essential part of the overall care management process that a United States nurse undertakes when caring for patients, especially in hospital settings where the majority of IENs work. If basic pathophysiology knowledge is poor, laboratory test interpretation skills will also be poor. Nevertheless, there are contextual factors in the case of IENs that may also influence performance in this area – if laboratory tests are not easily available for nurses to review as part of the care provided or if laboratory test interpretation for care planning is not considered part of the nursing role in their home country, then nurses would have little to no experience with it. Self-study or additional education would be required to overcome this specific exam performance gap.

Delegation of work is also a major function of the nursing role in the United States. Delegation, as a concept of measurement on the test, can include appropriate referrals to allied health professionals, such as physical therapy, and task delegation to licensed practical nurses and nursing assistants. It also incorporates concepts of leadership and advocacy (e.g. when to speak up for the patient when harm may occur). Nurses in many countries, especially LMICs, may not have allied health personnel to refer to nor a nursing assistant role. The passive role many nurses have in many countries, along with cultural dynamics related to gender, will influence what many nurses view as appropriate responses to questions. A good example of this kind of testing scenario is “What do you do in situation X if you cannot reach the physician?” For many IENs, the idea of not being able to reach a physician, or at least one in training, is not something they have experience with in their education or work. It is, however, a reality in nursing care delivery in the United States and critical to patient safety.

A secondary factor regarding the NCLEX-RN exam is that it was created to license nurses trained and educated to work in the United States and, more recently, Canadian healthcare systems. There is, therefore, an underlying bias towards nurses and nursing students trained within and familiar with the United States and Canadian systems. Because of poor work environments, severe human resources for health constraints and maldistribution of nurses, particularly in LMICs, nurses are facing high nurse to patient ratios and are limited in their capacity to provide safe, quality care; further, they do not have access to additional healthcare providers to delegate tasks [38–41]. Contextual experiences with care delivery, therefore, may affect pass rates given the variability in the perception of a correct answer.

The aforementioned curricular differences that affect IEN NCLEX-RN exam performance also have implications for the growing private education sector for health professions occurring in many countries. Many of these schools are designed to produce professionals for export and not necessarily to work in their home countries. While it would certainly be useful for nurses in any country to have a solid understanding of pathophysiology and laboratory test results, a nurses’ role is unique to their country of training and curricula reflect that. The changes in the NCLEX-RN exam may mean that schools designed to produce nurses for export to the United States are less likely than ever to succeed without some kind of transitional education program because there are contextually-specific role implementation issues that require training experiences in the country where the nurse will practice.

It is important to note that Canada has adopted the NCLEX-RN exam for its nurse credentialing requirements since 2015, although this does not include a French version of the exam for Quebec. Given the effects the NCLEX-RN exam has had on IEN pass rates in the United States as a mediating mechanism for IEN migration, it may do the same for Canada – another major receiving country of IENs. This may also shift migration dynamics from French speaking countries to Canada, many of which are in Sub-Saharan Africa and on the ‘do not recruit’ list.

It is important to note that even if an IEN passes the NCLEX-RN, it does not guarantee employment in the United States. Unless the education received was in English, IENs need to take an English language test with a spoken English component in order to obtain a work visa. Research conducted during the 2000s highlighted patient safety and satisfaction issues to be related to IENs’ English language level [28, 29, 36, 37], thereby providing evidence to justify the language test as part of the work requirements. Anecdotal reports suggest that IENs report being able to pass NCLEX-RN but not the general English language exam--sometimes even if their education was in English. This reduces the pool of eligible IENs for work in the US even further.

Nevertheless, for many providers and sending countries, the English language issue can be a double-edged sword. While nurses from non-English speaking countries who become proficient in English may cause alarm for some policymakers who think language skills might spur more  emigration, a point to consider is that the majority of evidenced-based practice and healthcare literature is published in English. Nurses with English language skills are more easily able to access evidence for practice to improve quality of care. Reading, writing, speaking, and listening skills can vary widely in a single individual and will influence their ability to pass the licensure exam. Incentivizing  nurses to learn English to enhance access to evidence-based practice to improve quality of care can safely be encouraged without promoting migration.

Regarding United States work visas, these have also remained stagnant since the 2008 economic crisis. IEN visas were allowed to expire as United States-educated graduates had difficulty finding jobs after the crisis [8]. With United States nursing workforce production now at replacement levels [14, 15], for the time being demand for IENs, unless they are Spanish speakers or speakers of other languages for which there is a high demand for language-concordant nurses, is low. In addition to passing the examination and language requirements, IENs also face financial, familial, and personal hurdles that may impede migration. Consequently, IENs with the intent to migrate and those that do may be disproportionate in numbers [37]. Additionally, IENs who do migrate may nevertheless decide not to practice. However, with the implementation of the Affordable Care Act, impending retirements from baby boomer generation nurses, and the silver tsunami of an aging population all slated to increase demand for healthcare services in the United States, demand for IENs could increase again in the next decade.

Finally, there are a number of limitations to consider regarding the present analysis. First, it is not possible to identify which IENs taking the exam were already living and working in the United States; nevertheless, these numbers tend to be small. Secondly, despite knowing that the overall data quality is sound given its sources, several methodological challenges may emerge in longitudinal analyses and the number of units involved in the current study. Lastly, concerns with regards to the model being underpowered were raised; however, the robustness checks adequately addressed this issue. Nonetheless, we plan to continue exploring these trends with additional years of data.

## Conclusion

The present study suggests that, while the WHO Code has had an influence on overall IEN migration dynamics to the United States by decreasing candidate numbers, in most cases, this was not the single source of variation. The impact of the NCLEX-RN exam changes and the current nature of the test appear to exert a larger influence, which, combined with the WHO Code, becomes even more powerful. While free trade advocates dislike credentialing exams for professionals since they view them as a barrier to trade [42–44], when people’s lives are at risk, an exam like the NCLEX-RN assures the public that at least a safe baseline level of knowledge and competency can be expected at the point of care from any nurse no matter where they received their education. The exam also provides equitable assessment of potential candidates since it is a requirement for all United States nurses; international candidates are therefore meeting the same standards as domestically-educated nurses. Concerns about discrimination due to country of origin, therefore, are minimized in the credentialing process. Countries or regions with high levels of migration seeking to manage IEN migration dynamics may want to consider credentialing exams as a migration mediation strategy.

A salient point for discussion emerging from these results is the United States’ role in the health worker ‘brain drain’ phenomenon, specifically as it relates to nurses. The United States is frequently listed as the top international destination of health workers and a major contributor to brain drain from LMICs. Nevertheless, with 69 % of the IENs originating from two countries and only 4 % of all candidates originating from 46 out of the 57 ‘do not recruit’ countries, the label (in terms of nursing) is questionable. Certainly, the argument could be made that the United States contributes disproportionately to the nursing brain drain from the Philippines and India, but the global effect argument weakens when considering the overall global picture of the phenomenon. Ethical recruitment policies in the United States, moving forward, may need to consider ways to balance recruitment among countries and work visa allocation strategies so that one single country is not disproportionately affected by voluntary migration or purposive recruitment practices.

To determine the future of the healthcare labour market for IENs, it is important to understand how the global economic crisis and the WHO Code have influenced migration patterns to the United States in the context of changing domestic labour market conditions. The interplay of these variables has the potential to influence future labour market demand for and policy strategies applied to IEN recruitment in the United States. Other high-income countries may find the study informative when designing their own international recruitment policies for nursing personnel.

## Abbreviations

GNI-PPP: Gross national income based on purchasing power parity
IEN: Internationally-educated nurse
LMICs: Low- and middle-income countries
NCLEX-RN: National council licensure examination-registered nurse
NCSBN: National council of state boards of nursing

## Footnotes

1 A forthcoming manuscript from Scheffler, R., J. Liu and T. Bruckner (forthcoming). Forecasting Global Health Workforce Supply, Demand and Needs to 2030. World Bank: Washington, DC will be publishing updated statistics regarding the global human resources for health shortage.

## References

[1] UN-DESA and OECD. World migration in figures. 2013.https://http://www.oecd.org/els/mig/World-Migration-in-Figures.pdf. Accessed 28 Jan 2015.

[2] Buchan J, Parkin J, Sochalski T (2003). International nurse mobility: Trends and policy implications.

[3] International Council of Nurses and Florence Nightingale International Foundation. The global shortage of registered nurses: An overview of issues and actions. 2005. http://www.icn.ch/images/stories/documents/publications/GNRI/The_Global_Nursing_Shortage-Priority_Areas_for_Intervention.pdf. Accessed 28 Jan 2015

[4] World Health Organization (2013). A universal truth: No health without a workforce.

[5] Taylor AL, Dhillon IS (2011). The WHO Global Code of Practice on the International Recruitment of Health Personnel: The Evolution of Global Health Diplomacy. Glob Heal Gov..

[6] Edge JS, Hoffman SJ (2013). Empirical impact evaluation of the WHO Global Code of Practice on the International Recruitment of Health Personnel in Australia, Canada, UK and USA. Global Health..

[7] World Health Organization (2011). User’s Guide to the WHO Global Code of Practice on the International Recruitment of Health Personnel.

[8] NCSBN. Exam Statistics & Publications. Practice Analyses. 2015.http://ncsbn.org/7569.htm. Accessed 28 Jan 2015.

[9] Gantz NR, Sherman R, Jasper M, Choo CG, Herrin-Griffith D, Harris K (2012). Global nurse leader perspectives on health systems and workforce challenges. J Nurs Manag.

[10] Buchan J, O’May F, Dussault G (2013). Nursing workforce policy and the economic crisis: a global overview. J Nurs Scholarsh.

[11] Buerhaus PI, Auerbach DI, Staiger DO (2007). Recent trends in the registered nurse labor market in the U.S.: short-run swings on top of long-term trends. Nurs Econ.

[12] American Associaton of Colleges of N. New AACN data show an enrollment surge in baccalaureate and graduate programs amid calls for more highly educated nurses. Press Release, 2012. http://www.aacn.nche.edu/news/articles/2012/enrollment-data. Accessed 28 Jan 2015. 10.1016/j.profnurs.2012.04.001 22812027

[13] American Association of Colleges of Nursing, Resarch and Data Center.. Qualified applications turned away from entry-level baccalaureate nursing programs: 2002–2013. 2013. Accessed 28 Jan 2015

[14] Auerbach DI, Buerhaus PI, Staiger DO (2011). Registered nurse supply grows faster than projected amid surge in new entrants ages 23–26. Health Aff. (Millwood).

[15] Health Resources and Services Administration (2014). The future of the nursing workforce: national- and state-level projections.

[16] Squires A, Beltrán-Sánchez H. Strengthening health systems in North and Central America: what role for migration ? Washington, DC; 2013. http://www.migrationpolicy.org/research/strengthening-health-systems-north-and-central-america-what-role-migration. Accessed 28 Jan 2015.

[17] Health Resources and Services Administration (2014). Technical documentation services administration’s health workforce simulation model.

[18] Lujan J (2008). Linguistic and cultural adaptation needs of Mexican American nursing students related to multiple-choice tests. J Nurs Educ.

[19] Starr K (2009). Nursing education challenges: students with English as an additional language. J Nurs Educ.

[20] Crossley SA, Salsbury T, McNamara DS (2012). Predicting the proficiency level of language learners using lexical indices. Lang Test.

[21] Croissant Y, Millo G (2008). Panel data econometrics in \proglang{R}: the \pkg{plm} package. J Stat Softw.

[22] National Council of State Boards of Nursing (2004). Number of candidates taking NCLEX examination and percent passing, by type of candidate.

[23] National Council of State Boards of Nursing (2005). Number of candidates taking NCLEX examination and percent passing, by type of candidate.

[24] National Council of State Boards of Nursing (2007). Number of candidates taking NCLEX examination and percent passing, by type of candidate.

[25] National Council of State Boards of Nursing (2008). Number of candidates taking NCLEX examination and percent passing, by type of candidate.

[26] National Council of State Boards of Nursing (2010). Number of candidates taking NCLEX examination and percent passing, by type of candidate.

[27] National Council of State Boards of Nursing (2011). Number of candidates taking NCLEX examination and percent passing, by type of candidate.

[28] Xu Y, He F (2012). Transition programs for internationally educated nurses: what can the United States learn from the United Kingdom, Australia, and Canada?. Nurs Econ.

[29] Xu Y (2007). Strangers in strange lands. Adv Nurs Sci.

[30] Xu Y (2007). International migration of nurses and human development. Home Health Care Manag Pract.

[31] Edwards PA, Davis CR (2006). Internationally educated nurses’ perceptions of their clinical competence. J Contin Educ Nurs.

[32] Mehta SD, Hall J, Lyss SB, Skolnik PR, Pealer LN, Kharasch S (2007). Adult and pediatric emergency department sexually transmitted disease and HIV screening: programmatic overview and outcomes. Acad Emerg Med.

[33] Kawi J, Xu Y (2009). Facilitators and barriers to adjustment of international nurses: an integrative review. Int Nurs Rev.

[34] Thekdi P, Wilson BL, Xu Y (2011). Understanding post-hire transitional challenges of foreign-educated nurses. Nurs Manage.

[35] Higginbottom GM (2011). The transitioning experiences of internationally-educated nurses into a Canadian health care system: a focused ethnography. BMC Nurs..

[36] Xu Y (2010). Is transition of internationally educated nurses a regulatory issue?. Policy Polit Nurs Pract.

[37] Xu Y (2010). Transitioning international nurses: an outlined evidence-based program for acute care settings. Policy Polit Nurs Pract.

[38] Hongoro C, McPake B (2004). How to bridge the gap in human resources for health. Lancet.

[39] Chen L, Evans T, Anand S, Boufford JI, Brown H, Chowdhury M (2004). Human resources for health : overcoming the crisis. Lancet.

[40] Araújo E, Maeda A (2013). How to recruit and retain health workers in rural and remote areas in developing countries: a guidance note.

[41] Lehmann U, Dieleman M, Martineau T (2008). Staffing remote rural areas in middle- and low-income countries: a literature review of attraction and retention. BMC Health Serv Res..

[42] Adlung R, Carzaniga A (2001). Health services under the General Agreement on Trade in Services. Bull World Health Organ.

[43] Mattoo A, Mishra D (2009). Foreign professionals in the United States: regulatory impediments to trade. J Int Econ Law.

[44] Clemens M. A labor mobility agenda for development working paper 201. Washington DC; 2010. http://www.cgdev.org/publication/labor-mobility-agenda-development-working-paper-201

